# How to measure the need for transition to adult services among young people with Attention Deficit Hyperactivity Disorder (ADHD): a comparison of surveillance versus case note review methods

**DOI:** 10.1186/s12874-019-0820-y

**Published:** 2019-08-20

**Authors:** Helen Eke, Astrid Janssens, Johnny Downs, Richard M. Lynn, Cornelius Ani, Tamsin Ford

**Affiliations:** 1 University of Exeter Medical School, South Cloisters 1.01, St Luke’s Campus, Exeter, EX1 2LU UK; 2 Child and Adolescent Psychiatry Surveillance System, London, UK; 3 Kings College London, De Crespigny Park, Denmark Hill, London SE5 8AF UK; 4 British Paediatric Surveillance Unit, Royal College of Paediatrics and Child Health, 5-11 Theobalds Rd, London, WC1X 8SH UK; 5 Surrey & Borders Partnership NHS Foundation Trust, Redhill, UK; 6 Centre for Psychiatry, Imperial College London, 7th Floor Commonwealth Building, Du Cane Road, London, W12 0NN UK; 7 User Perspectives, University of Southern Denmark, DK-5000 Odense C, Denmark

**Keywords:** Surveillance, CAPSS, CRIS, Case note review, ADHD, Transition, NICE

## Abstract

**Background:**

Health services have not provided adequate support for young people with long term health conditions to transfer from child to adult services. National Institute of Health and Care (NICE) guidance on transition has been issued to address these gaps. However, data are often sparse about the number of young adults who might need to transition. Using Attention Deficit Hyperactivity Disorder (ADHD) as an exemplar, this study used an existing surveillance system and a case note review to capture the incidence of the transition process, and compared and contrasted the findings.

**Methods:**

The Child and Adolescent Psychiatry Surveillance System (CAPSS) was used to estimate the incident transition of young people with Attention Deficit Hyperactivity Disorder (ADHD) from child to adult services. This involves consultant child and adolescent psychiatrists from the United Kingdom (UK) and Republic of Ireland (ROI) reporting relevant young people as they are seen in clinics. In parallel, a case note review was conducted using the Maudsley Biomedical Research Centre (BRC) Clinical Records Interactive Search (CRIS). The study period ran for twelve months with a nine month follow up to see how the transition proceeded.

**Results:**

CRIS identified 76 cases in the study period, compared to 18 identified using surveillance via CAPSS. Methodological issues were experienced using both methods. Surveillance issues; eligibility criteria confusion, reporting errors, incomplete questionnaires, difficulties contacting clinicians, and surveillance systems do not cover non-doctors and psychiatrists who are not consultants. Case note review issues using CRIS included the need for researchers to interpret clinical notes, the availability and completeness of data in the notes, and data limited to the catchment of one particular mental health trust.

**Conclusions:**

Both methods demonstrate strengths and weaknesses; the combination of both methods in the absence of strong routinely collected data, allowed a more robust estimate of the level of need for service planning and commissioning.

## Background

Several studies, government documents and policy guidelines highlight the difficulty that young people face who require a transition from child to adult services [1]. Transition between services is particularly difficult for young people with Attention Deficit Hyperactivity Disorder (ADHD) [1]. Two types of transition can be conceptualised; *developmental* when a child moves from adolescence to adulthood, and *situational*, moving from one service to another [1]. In the current paper, the term transition refers to a situational transition between child and adult mental health services, however this is driven by young people reaching a developmental transition. There is currently limited evidence available on the need for transition between services for young people with long term health conditions who require ongoing care. A case note review study would most commonly be used to capture the transition process; however it suffers from limitations such as requiring intensive researcher or clinician time, being very localised in focus, and the availability and quality of data are dependent on the clinician who records it [2–4]. This paper reports on two existing methods that have been adjusted to estimate the national need for young people with ADHD to transition to adult services; they were a surveillance system and an electronic case note review method. Paediatric surveillance studies have traditionally focussed on the incidence of rare conditions [5], however the current study focussed on the incidence of transition between child and adult services for young people with ADHD as an *event or process* as opposed to the incidence of ADHD as a *condition*. ADHD itself is not rare; it is one of the most common long term conditions managed by child and adolescent mental health service (CAMHS) and community paediatric services [6].

The prevalence of ADHD is estimated at approximately 5% [7], and population based studies suggest that 15% of those with childhood ADHD still meet the full diagnostic criteria for the disorder at age 25 [8]. However, existing research suggests a seamless transition process between child and adult services happens much less often than can be expected based on adult prevalence rates [9]. Two previous studies [1, 10] have reviewed case notes narratively to identify transition cases between CAMHS and adult mental health services (AMHS) over a twelve month period. The first study identified an average of 12 cases of neurodevelopmental disorder per CAMHS team that were eligible for transition in one year, but 40% were not referred to any adult service, and only 67% of those referred actually made the transition [1, 11]. The study was based on a limited number of health trusts in England and identified neurodevelopmental cases in general, not ADHD specifically. The second study focussed on ADHD cases in Ireland, and identified 20 patients from four CAMHS teams that required a transition. No cases were directly transitioned to AMHS; they were either retained by CAMHS, referred to a private service, or discharged to their General Practitioners (GP) [10]. Extrapolating from epidemiological studies can be helpful in the estimation of population level need, but does not necessarily provide information about service access and service-level need, and may not be relevant to populations other than those studied. An existing prospective North American longitudinal study used assessment at three time points from age 9 to 30 years, as a method to quantify patterns of transitions, and it was found that ADHD in particular showed a strong continuity across the transition from adolescence to adulthood [12]. Studies of long term conditions such as ADHD rarely follow participants across developmental transitions [13] and national empirical data on the number of young people that wish to access ongoing care for ADHD in adulthood, or the number that successfully and seamlessly access follow up care in early adulthood, is sparse. This hampers commissioning and provision of services for this group.

The current paper describes how two existing methods were adjusted to assess the need for transition between child and adult services for those with ADHD at a national level; it evaluates how feasible and transferable these methods were to quantify and capture the need for transition or other rare events or processes in health services. To estimate incident service need for young people with ADHD to transition to an adult service we used a paediatric surveillance methodology, in particular the Child and Adolescent Psychiatry Surveillance System (CAPSS), and an electronic clinical case note search using the Maudsley Biomedical Research Centre (BRC) Clinical Records Interactive Search (CRIS) [14].

## Methods

This study was part of a larger National Institute of Health Research (NIHR) funded project on transition for young adults with ADHD [15], which included a surveillance study using the British Paediatric Surveillance Unit (BPSU) and CAPSS simultaneously. For the purposes of this paper, CAPSS and CRIS are discussed independently from the wider NIHR study.

### Surveillance methodology

The CAPSS surveillance system was used to collect data on incident transition. This surveillance system provides a coordinated data collection system; it is designed to collect notification reports from clinicians, and to support research teams to gather data from the clinicians about each patient, with follow up to assess outcome and understand management [16]. The methodology was developed by BPSU and is a well-established and very successful system traditionally used to study rare paediatric disorders and events across the United Kingdom (UK) and Republic of Ireland (ROI) without selection bias. The system has been replicated around the world for paediatric surveillance, but also for different specialities [17]; CAPSS is one example and collects notifications from Consultant Child and Adolescent Psychiatrists [18].

**Fig. 1:**
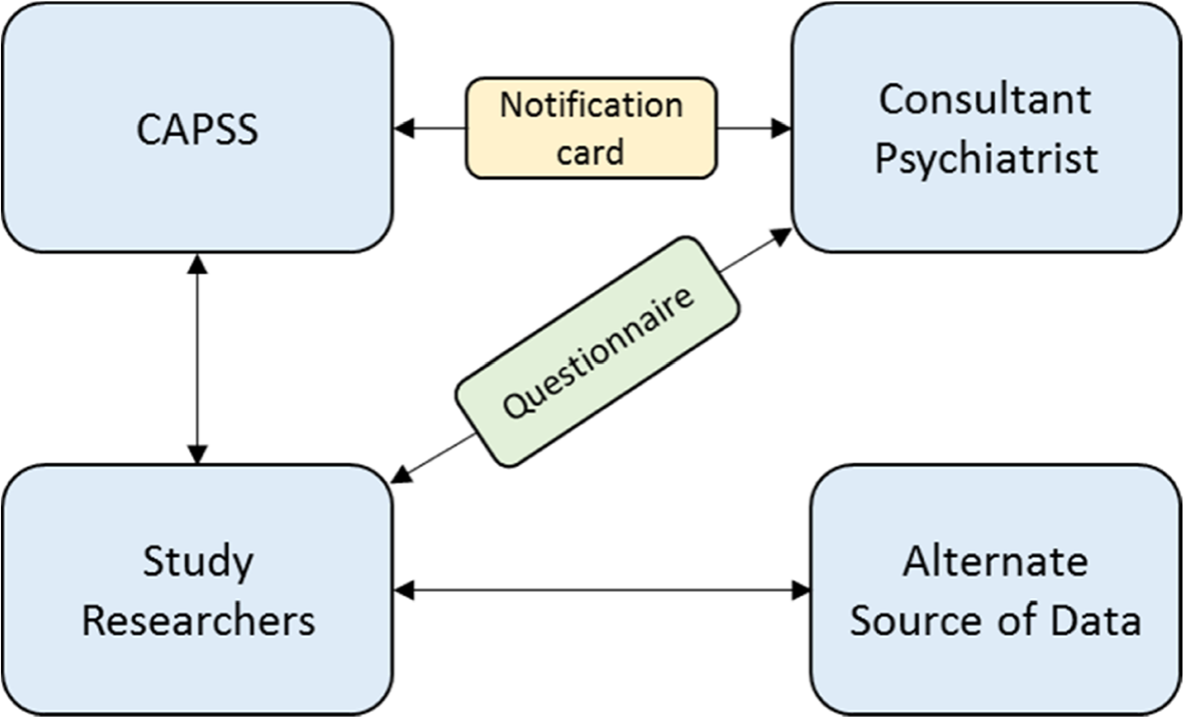
Surveillance Methodology

Figure 1 illustrates the surveillance process. Approximately 1000 consultant child and adolescent psychiatrists are sent a surveillance ‘reporting card’ (now 90% via email) each month in order to report uncommon disorders to all current listed research studies. More details on the process are described elsewhere [19–21].

Relevant ethical approval was obtained. The governance structure for surveillance studies is complex, challenging and fluid. Surveillance data is not publically available and this type of surveillance required Health Research Authority (HRA) approval as cases may be reported from any NHS Trust that works with children across the UK. In addition, Section 251 approval is required from the Confidentiality Advisory Group (CAG) to permit clinicians to report anonymous case note information without patient/parent consent provided there is no requirement or expectation for additional patient contact as a result of the study. (HRA-IRAS reference 159209, CAG reference 15/CAG/0184).

CAPSS is set up to investigate rare childhood disorders [21] defined as less than 1:20,000 incidence. Thus, conditions are accepted for study based on rarity and public health relevance. ADHD is not a rare condition, yet the successful transfer of care for a young person with ADHD between child and adult services is recognised as uncommon and of relevance to both paediatricians and child and adolescent psychiatrists [9]. The first month of any surveillance study is treated as a pilot to iron out any difficulties with definitions and because prior experience indicates that prevalent cases are often reported due to interest about the study. CAPSS recognised the public health relevance of monitoring this event, but expressed concerns that this could result in large numbers of cases (more than 360 per year) which would overload the system [19, 22]. The study was initially approved for six months active surveillance (half the time period of typical surveillance studies) with the option to extend to a full year depending on the number of cases reported. In total the surveillance study ran for thirteen months from November 2015 to November 2016, which included the first pilot month. The follow up period ran from August 2016 to August 2017, and was at nine months for each reported case.

#### Case definition criteria

This study was the first time that a surveillance case definition had described a health service process and not just a condition checked by a review of clinical symptoms. The surveillance definition was very different to usual surveillance criteria [23]. In addition, as this study was part of a larger project, the definition had to be appropriate for both paediatricians and psychiatrists, to ensure that both sets of clinicians would identify the same transition processes. Therefore, the case definition was designed in close collaboration with members of BPSU and CAPSS, and required repeated iterations involving stakeholders from an advisory group, and both BPSU and CAPSS scientific committees. The definition had to be agreed before the application for the study could be approved. The first pilot month also allowed for any issues with the case definition to be raised.

Table 1 lists the case definition criteria that had to be met in order for a case to be eligible for inclusion in the study.

**Table 1:** Surveillance Case Definition Criteria

1. A young person with a clinical diagnosis of ADHD under the care of CAMHS, who is reviewed for the first time when, within 6 months of reaching the services’ age boundary, whatever this may be. Young people should only be reported once and those that have already been seen and reported in this time-scale should not be reported a second time.	
2. The young person is considered to require continued drug treatment for their symptoms of ADHD after crossing the service age boundary.	
3. The young person should not have been reported previously to the BPSU/CAPSS in relation to the current study.	
4. A young person with ADHD and comorbid diagnoses, including learning / developmental disabilities, should be reported only if it is their ADHD for which on-going drug treatment in adult services is required.	

The case definition criteria were developed to be precise and clearly defined, and to specify the ongoing need for support from specialist adult mental health services as concretely as possible, while echoing the recommendations outlined in the NICE guidelines for transition in health and social care services [24]. The aim of the case definition was to provide a minimum estimate of the number of young people with ADHD who required a transfer from CAMHS or paediatrics to adult services during the surveillance period. As different CAMHS and Adolescent services are likely to have different protocols and service specifications, and there are also different types of services available to treat young people with ADHD such as 0–25 services, the age boundary was unspecified in the case definition in order to measure when the transition was actually occurring as a secondary aim. Requirement for ongoing medication was chosen as a criterion in order to rule out subjectivity in the application of definitions of ‘ongoing care’. It would not capture those who did not need or want medication but did need ongoing psychological support.

#### Questionnaire development

Baseline notification and follow up questionnaires were developed according to guidance for developing a surveillance study [22], and each questionnaire was highly structured and as brief as possible. The baseline questionnaire was sent to all clinicians that reported a case to the study. The questions confirmed eligibility, and collected sufficient patient data to detect and remove duplicates. It also collected details of patient treatment, and details of the planned transition to an adult service. Any professional with access to the patient notes could complete the questionnaire on behalf of the consultant, but the notification card and report were always sent to the consultant child and adolescent psychiatrist via the relevant surveillance unit. A nine month follow up questionnaire was sent to the same reporting clinician to confirm the outcome and details of the transition. Email and postal reminders for non-returned questionnaires were sent after 4 weeks and after 6 weeks. Finally a follow up telephone call was made if the questionnaire was still outstanding.

### Case note review

The electronic clinical case note system from the Maudsley Biomedical Research Centre (BRC) Clinical Record Interactive Search (CRIS) at the South London and Maudsley (SLaM) NHS Foundation Trust, was chosen as the system for the case note review methodology. CRIS is not publically available, but provides authorised researchers access to secure, regulated, anonymised patient data extracted from electronic clinical patient notes [14]. Data from CRIS were used over the same time period, applying the same criteria as the surveillance study, to identify cases. Comparison could only be drawn against a subset of the data collected using CAPSS, as SLaM provide mental health services only. CRIS was approved as an anonymised data resource for secondary analysis by Oxfordshire Research Ethics Committee (08/H0606/71 + 5). This project was reviewed and approved by the CRIS patient-led oversight committee (CRIS project ref.:961).

The case definition criteria from the surveillance study was operationalised into a structured query language (SQL), which was used to identify relevant cases in CRIS. This search produces an output of anonymous electronic records that meet the search criteria. Manual review of the electronic records by two researchers extracted the individual, clinical and service related characteristics of the case, including any available details about transition (see Table 2). Two researchers were used as the process was time intensive. It also prevented bias that might occur from a single researcher; approximately 50% of the case note records were double screened.

**Table 2:** Complete list of CRIS data outputs extracted for data collection

CRIS ID	Reason for appointment	Other medication 3
Gender	CAMHS or AMHS	Other medication 4
Ethnicity	Seen by Clinician	CGAS score 1–100
DOB (specified)	Comorbidity 1	SDQ Assessment Date
Truncated Postcode	Comorbidity 2	SDQ total score
Social Deprivation	Comorbidity 3	Hyperactivity score
Date of Diagnosis of ADHD	Comorbidity other	Impact score
CAMHS Directorate	ADHD Medication 1	Contact frequency
Last date seen	ADHD Medication 2	DNA rate

### Analysis of data from surveillance and case note review

A measure of compliance with a surveillance scheme is the proportion of reporting cards returned [25]. The successful utility of the surveillance methodology is discussed by exploring the use and understanding of the case definition, errors with reporting and the case definition, compliance to monthly reporting cards and return of questionnaires, and the mechanisms of the surveillance databases used.

Data linkage and protection governance meant that cases from CRIS could not be directly linked to the patient data collected in the surveillance study, thus only estimates and descriptive characteristics of cases captured by the two methods could be compared. It was also not possible to directly replicate the same boundary of SLaM in the CAPSS data as researchers are blinded to identifiable patient data and the information provided on each case related to the reporting consultant and not the service or clinic. The wider boundary of ‘London’ was used in the CAPSS data to compare against.

The incidence of transition collected via both systems was compared. Data from each source were extracted by a researcher and collated in to a spreadsheet using Excel. Descriptive data were collated for the number of cases identified, gender, ethnicity, and the reviewing clinician. Further descriptive data were also collated for transition referral date, referral acceptance, first appointment in adult service, evidence of joint meetings and persons involved in transition. These were tabulated and directly compared.

## Results

### Overall acceptance of surveillance methodology

Over the twelve month period, there was a mean response rate to the CAPSS monthly reporting cards of 53% (total of 7016 cards sent). This is lower than CAPSS have reported previously [18, 26]. In total there were 300 CAPSS case notifications; more cases than existing studies on transition have suggested in one year [1, 10, 11]. The response rate to the questionnaires (58% at baseline and 83% at follow up) was also slightly lower than reported by CAPSS in other studies [17, 26]. Further descriptive results of the surveillance data collected are discussed in a separate paper [27]. For the purposes of this paper, only the CAPSS cases that were identified to be reported from within the boundary of London (*n* = 45) were included in the analysis.

#### Surveillance case definition

The case definition raised some issues, particularly at the beginning of the surveillance period. Misunderstanding occurred around the term ‘*reviewed for the first time, within six months of the age boundary’*. It was unclear to some clinicians if it was the first time they had ever reviewed the patient, and thus a new diagnosis, or whether it was first time reviewed during the surveillance period. Queries were resolved directly with the clinician by explaining the terminology as ‘*the first time the case is seen during the surveillance period*’. Most errors that occurred due to this confusion occurred during the first pilot month; these data were excluded from subsequent analysis as per protocol.

Other errors in reporting from clinicians included; two consultants who reported a whole caseload of ADHD patients rather than reporting just the patients that required a transition and were at transition age; five cases were reported more than once as they were seen more than once during the study period (this was the reason for the “reviewed for the first time” criterion); consultants who were not able to remember the patient details when asked to complete the baseline questionnaire (*n* = 16); cases that did not meet one or more of the five case definition criteria (*n* = 30); and ‘reporting in error’ e.g. ticking wrong box on card, reading the card incorrectly, no recollection of reporting (*n* = 19).

#### Surveillance data collection

Registration with CAPSS is voluntary and therefore not all consultants may be registered to receive the reporting cards. Only consultant and associate specialist level clinicians are enrolled once identified; other clinicians may review patients with ADHD at the transition boundary, particularly if they are clinically well, but would not be contacted to notify to CAPSS. Some contact details provided by the surveillance organisation was out of date (*n* = 8, 7%), which prevented the research team reaching the clinician with the questionnaire. The research team made alternative efforts to reach the clinician, for example with help from CAPSS, or by using search engines and contacting the clinic or hospital directly.

The response rate to questionnaires was in line with results of other surveillance studies, but there was no response at all from 42% of questionnaires (*n* = 127), some were returned blank (*n* = 1) or with missing data (*n* = 39, 13%) and it was reported anecdotally as time intensive to complete (*n* = 6, 2%). The sections most frequently left blank at baseline were the facts regarding transition, for example what service the patient was referred to. At follow up the questions most frequently left blank were the elements of optimal transition, for example whether a joint meeting between services took place. Efforts were made to contact clinicians directly to complete any missing data.

### Comparison of methods

Table 3 compares the CAPSS surveillance and the case note review data collected using CRIS. The CRIS database identified 91 ADHD cases in SLaM who had a clinical diagnosis of ADHD, were within six months of the service age boundary, and therefore potentially eligible for transition. However, there was evidence in the case notes that 15 cases were discharged prior to transition or were no longer on medication or requiring treatment, leaving 76 that met all of the study criteria. There were 45 CAPSS cases identified based on the location of the reporting clinician, 18 of which were confirmed as eligible cases from the baseline questionnaire.

**Table 3:** CAPSS-CRIS comparison

	CAPSS	CRIS
Notifications/Identified cases (n)	45	91
Did not meet eligibility criteria (n)	27	15
Met all eligibility criteria (n)	18	76
Eligible cases only		
Gender ratio (m%:f%)	83:17	84:16
Ethnicity (% White British)	72	46
Reported/Reviewed by Consultant (n)	18	41
Reported/Reviewed by other health professional (n)	0	35
Transition referral made, accepted and first appointment offered in adult service (n)	10	37
First appointment confirmed as attended (n)	4	28

All CAPSS cases were reported by a consultant level clinician, while only half (54%, *n* = 41) of the 76 eligible CRIS cases were reported to have been seen by a consultant. The remaining 46% of cases were either seen by an alternative health professional or the case notes did not indicate who had reviewed the patient.

## Discussion

A well-established surveillance method, and a case note review method have been used to assess the need for, and process of, transition between child and adult services for young people with ADHD. Utilised together, both methods have provided an insight into the need for transition of patients with ADHD in CAMHS settings in the UK.

CAPSS was chosen in order to most closely reflect the common health setting that patients with ADHD are reviewed in within the UK, and the monthly notification card completion rate throughout the study, which was similar to the average CAPSS rates, shows that this method of surveillance is successful. It also has the potential to be used to monitor a health service event seen by psychiatrists, in addition to incidence of rare conditions which it is traditionally used for. CAPSS had the potential to provide a national picture, in contrast to the case note review that is localised. When compared to the clinical case note review via CRIS, there was a significant difference in the number of cases identified using CAPSS. However, the case note review method allowed all cases to be captured within a NHS trust that required transition due to ongoing medication needs, regardless of the treating or supervising clinician, whereas surveillance relies on the clinician to accurately report each case.

Using CAPSS, only consultant level psychiatry clinicians are sent reporting cards each month. The case note review with CRIS demonstrated clearly that ADHD patients in this mental health trust are frequently reviewed by other health professionals, such as junior doctors and specialist nurses. These clinicians would not be able to report to CAPSS. Similarly locum clinicians also may not be registered with, or reporting via CAPSS due to frequent employment moves. Even if locums are registered and do notify cases, they may have moved posts between notification and baseline questionnaire, or between baseline and follow up, and therefore not be able to provide data. Patients may also be reviewed in settings other than CAMHS, such as primary care or forensic services [28], while a study of surveillance approaches has highlighted the absence of surveillance in the private sector despite it playing an important role in health care provision [29]. This is perhaps particularly relevant for young people with ADHD for whom there can be long waits for treatment in the public sector and gaps in the provision of adult services [15, 30].

Enrolment with CAPSS is voluntary, and therefore not all consultants may be registered to receive the reporting cards. A census in 2017 reported there to be 5395 registered consultant psychiatrists (approximately a quarter of which are child and adolescent psychiatrists), whilst CAPSS have approximately 1000 on their database [31–33]. It is possible that although clinicians are registered with CAPSS, they may be academics and not hold relevant clinical caseloads, and would therefore not be reviewing patients with ADHD. Some contact details provided by the surveillance organisation were out of date, inevitably due to clinicians frequently changing NHS Trusts, location, role, retiring, or working as locum. Incomplete records present a factor in non-return despite efforts to update records and the use of alternative methods to contact clinicians.

The surveillance system relies wholly on accurate reporting from the clinician, and the average return rate of the reporting cards was low in CAPSS when compared to previous BPSU studies [17]. The BPSU system was adopted and replicated by CAPSS in 2009, and perhaps it is yet to become routine practice for child and adolescent psychiatrists. The lower return rate of notification cards may indeed indicate a lack of awareness of the system and not necessarily a reflection of clinicians actively being non-compliant. It is possible that the difficulties with the case definition could also have led to a lack of reported cases. Previous surveillance studies have also cited difficulties with reporting, case definitions and lower return rates [26, 34–36]. Clinicians were offered certificates to demonstrate time committed to research to be used for appraisal, as an incentive to return questionnaires to the study. There is no evidence to suggest that this had an impact on return of questionnaires, however certificates were generally received with thanks.

It is important to estimate the accuracy of case ascertainment in surveillance [37] and verify findings [38]. It is recommended that researchers conducting surveillance studies reconcile their data with other sources to help improve completeness and accuracy [16]. Previous surveillance studies [20, 39, 40] have used ‘capture recapture’ analysis to maximise case ascertainment, but for this matched cases must be identifiable and the population under study must be closed [20]. This was not possible in the current study of transition in ADHD. The CRIS database was used as an alternative data source to estimate the incidence of transition among young people with ADHD and to compare against the data collected using surveillance.

There were limitations to this comparison. While clinicians completing the surveillance questionnaires had direct knowledge of the young people as well as the case notes that they themselves may have written, the data collected using CRIS relied on information recorded by other people in the clinical notes [38]. Inevitably, this involved some subjective judgements on behalf of the researchers as the clinical notes may not necessarily include readily available concrete information such as prescribed medication, comorbidities or details of diagnosis. Similarly, the information gathered by the surveillance questionnaires was specified by the study researchers, and required short, succinct responses. It was not always possible to identify the same succinct information from the clinical case notes.

While the surveillance system has the advantage of its national cover, the case note review was limited to a single NHS trust covering one part of a metropolitan city. How representative these services and the young people attending them are of all young adults with ADHD is difficult to judge. Existing research has alluded that patients identified in case registers are not always representative of all cases with that disorder [3]. Importantly, and a key limitation of this study is that the geographic location of SLaM clinics and hospitals could not be directly replicated in the surveillance data, as the address provided from the surveillance notifications was that of the clinician, and not of the clinic or hospital in which the patient was seen. The broader term of ‘London’ was used in the CAPSS data which almost certainly gathered cases from a wider boundary than is included in SLaM. There are nine mental health trusts in London, of which eight have CAMHS services [41] (SLaM is one of them), suggesting that the data collected in CRIS only represents a fraction of the ADHD transition cases in London.

Data protection and information governance meant that data could not be directly linked which would have allowed more direct inference of the completeness of case ascertainment from CAPSS to be drawn. Interestingly, data protection rules may be more stringent than the attitudes of many patients and public. A previous study has highlighted the benefits of linking data to provide information that is missing and reduce bias [42] and a study of attitudes towards linking data concluded that it was perceived acceptable to share health data in a medical context [43]. A study using medication registers has previously been used to examine trends over transition [44], but not all trusts have such registers, and prescribing for ADHD is often led by primary rather than secondary health care.

While the case note review clearly provides the most efficient local data, both methods offer strengths and weaknesses in terms of our attempt to provide robust national estimates. While imperfect, these results, particularly when combined, provide an insight in to the issue of transition for young people with ADHD nationally that has not been achieved by studies previously. Ideally, routine data linkage could inform service planning and provision at national and local levels in real time, but whilst systems like CRIS are located within a limited number of trusts, additional methods will be required. Many data protection and information governance issues currently mean that access to such data is difficult to obtain when it exists.

Previous research has suggested that traditional public health approaches for monitoring incidence of conditions is too late, too costly and often inaccurate [45]. Managing and running a surveillance study is labour intensive, both from the perspective of the surveillance organisation and from the individual study team, but it provides value for money as research studies can be conducted simultaneously [5] and data can be gathered on a national level. It is worth noting that case note review, even using an isolated system such as CRIS, is also labour intensive if the questions asked requires active data extraction. For most conditions, surveillance studies are still the only source of national data [46]. The existing surveillance organisations stress that studies should not generate more than 360 cases per year [22]; for valuable national surveillance to be effective for more than just incidence of rare conditions, and to continue to inform public health policy [46], these systems need to be properly funded and supported to enable large scale national studies to be carried out. Previous research has highlighted that national data are not necessarily sufficient to address gaps and advance knowledge; the establishment of the international network of paediatric surveillance units (INOPSU) and the replication of the methodology in certain specialities, potentially provides methodological opportunities for researchers to gather invaluable data on uncommon conditions or health service events internationally [47] that should be further explored.

## Conclusion

This is the first study to survey the incidence of ADHD transition using an existing surveillance methodology, and the first study to directly compare and contrast the feasibility of two existing methods (surveillance and electronic clinical case note review) in quantifying the need for transition. Both methods offer different strengths and weaknesses. The application of the combination of both methods, as conducted in this study, provided an insight in to the transition of care for young people with ADHD at a national level, and suggests further work is needed to refine the methodologies in order to ensure that future such estimates are more robust.

## Data Availability

The datasets generated and/or analysed during the current study are not publicly available because they are part of ongoing research that is not yet published, but they are available from the corresponding author on reasonable request.

## Abbreviations

ADHD: Attention Deficit Hyperactivity Disorder
AMHS: Adult mental health services
BPSU: British Paediatric Surveillance Unit
BRC: Biomedical Research Centre
CAG: Confidentiality Advisory Group
CAMHS: Child and adolescent mental health services
CAPSS: Child and Adolescent Psychiatry Surveillance System
CATCh-uS: Children and Adolescents with ADHD in Transition between Child and Adult Services
CRIS: Clinical Records Interactive Search
HRA: Health Research Authority
SLaM: South London and Maudsley NHS Trust
